# Deglutition Syncope Due to Exaggerated Vagal Reflex

**DOI:** 10.7759/cureus.16005

**Published:** 2021-06-28

**Authors:** Xuanzhen Piao, Michael J Chaney, Grace W Ying, Artem Sharko, Shirly Samuel

**Affiliations:** 1 Internal Medicine, Chicago Medical School Rosalind Franklin University of Medicine and Science, North Chicago, USA; 2 Internal Medicine, Chicago Medical School Internal Medicine Residency Program at Northwestern Medicine McHenry Hospital, McHenry, USA

**Keywords:** swallow syncope, deglutition syncope, bradyarrhythmia, vagal reflex, pacemaker, unexplained syncope

## Abstract

Swallow or deglutition syncope is an uncommon cause of syncope associated with bradyarrhythmia and hypotension during food swallowing. Early recognition of this condition is imperative but challenging. We report a case of a 60-year-old female who presented with a complaint of intermittent lightheadedness after swallowing food. An episode of presyncope was observed and a reduced pulse rate from baseline was noted when she was instructed to eat a candy bar in the clinic. Further workup revealed normal in-office electrocardiogram, bilateral carotid ultrasound, transthoracic echocardiogram, and videofluoroscopic swallow study. Our goal in presenting this case is to raise awareness of the condition in medical literature and provide a good understanding of its clinical manifestation to prevent life-threatening events.

## Introduction

Swallow or deglutition syncope (DS) is an extremely rare type of neural-mediated reflex syncopal syndrome associated with bradyarrhythmia and hypotension in relation to food ingestion [1]. Since the first reported case in 1793, there have been only about 100 cases recorded in the literature to date [2-4]. These patients usually present with either presyncopal symptoms or evident syncope [3]. DS has been described to be associated with various esophageal disorders such as esophageal spasm, stricture, achalasia, hiatal hernia, and esophageal cancer [4-7]. In addition, about 15% of reported cases on DS were associated with cardiac diseases and approximately 39% of cases had unknown etiologies [8,9]. DS has also been reported in patients without underlying laryngopharyngeal, esophageal or cardiac conditions [10-12]. Recognition of this syndrome is often challenging due to overlapping symptoms with other medical conditions and low case volume. In this case report, we present a patient with swallow syncope who had Schatzki’s ring status post repair two years prior to presentation.

## Case presentation

The patient is a 60-year-old female with a past medical history of chronic gastroesophageal reflux disease, esophageal spasm, Schatzki’s ring status post repair two years prior to presentation, asthma, and mild mitral valve prolapse. She presented to the clinic with a complaint of reproducible episodes of lightheadedness immediately after swallowing food for the past four months with each episode lasting 10-60 seconds. She stated that swallowing any hard solid food could trigger these episodes, which almost always resolved spontaneously except for a few times where she was close to passing out. She denied any history of syncope, seizure, stroke, transient ischemic attack, myocardial infarction, cardiac arrhythmia, pulmonary embolism, orthostatic hypotension, ataxia, or vertigo. In regard to the review of symptoms, she denied fever, unintentional weight loss, headache, visual disturbance, neck pain, chest pain, shortness of breath, choking sensation, dysphagia, odynophagia, dyspepsia, nausea, vomiting, abdominal pain, or anxiety. She had appropriate fluid intake and denied any recent illness, head trauma, medication change, or dietary change. Her home medications included pantoprazole and an albuterol inhaler. She denied using tobacco or recreational drugs but endorsed drinking 3-5 servings of wine or beer per week.

**Figure 1:**
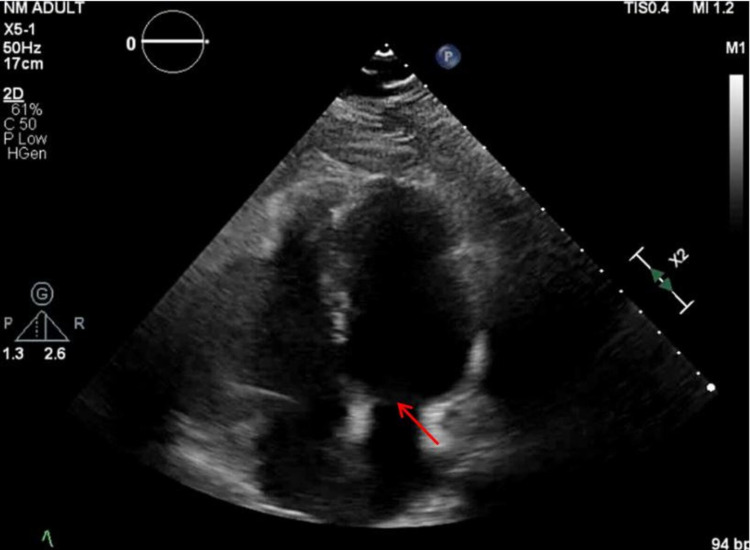
Two-dimensional TTE with Doppler in four-chamber view showing normal structure of the heart. There is no evidence of left ventricular hypertrophy. The left ventricular systolic function is normal with an estimated ejection fraction of 60%-65%. The trivial to mild mitral valve prolapse (arrow) remains stable compared to prior study. TTE - transthoracic echocardiogram

**Figure 2:**
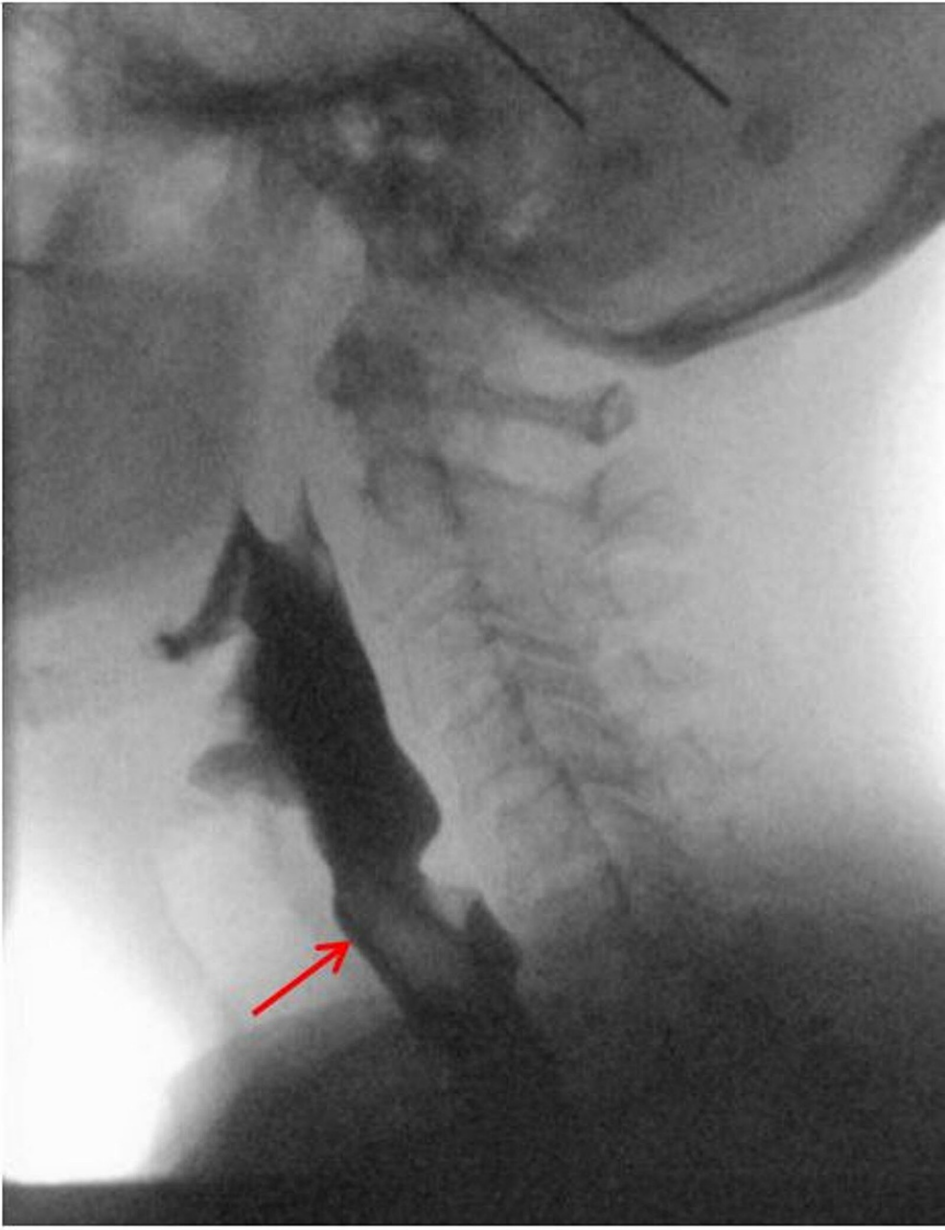
VFSS assessed the oral, pharyngeal, and esophageal phases of swallow. The figure shows honey-thick material coating the esophagus (arrow) demonstrating normal appearance without stricture or dysmotility. VFSS - videofluoroscopic swallow study

On presentation, she had a blood pressure of 126/88 mmHg, heart rate 97 beats per minute, temperature 98.1°F, respiratory rate 14 breaths per minute, and oxygen saturation of 99% on room air. The physical exam was unremarkable except for a mitral valve click that was heard over the left sternal border. The initial lab work including complete blood count and the comprehensive metabolic panel was unremarkable. During the initial visit, an episode of presyncope lasting about 10-15 seconds was observed and a reduced pulse rate from baseline was noted when the patient was instructed to eat a candy bar. She reported an intense feeling of lightheadedness but recovered spontaneously without confusion. She was told to continue to monitor her symptoms and a cardiology referral was given for further evaluation. Subsequent diagnostic workup revealed normal in-office electrocardiogram (EKG), bilateral carotid ultrasound, transthoracic echocardiogram (TTE) (Figure 1), and videofluoroscopic swallow study (VFSS) (Figure 2). The patient reported of having mild lightheadedness with swallowing a larger bolus of food during the VFSS, but there was no evidence of esophageal dysmotility or change in bolus drive, direction or residue. At home, she continued to experience lightheadedness with swallowing, but not to an extent of affecting her quality of life. Diet modification, symptom monitoring, and fall precaution were discussed and recommended to the patient. If symptoms were to worsen, the patient was instructed to return for a 24-hour ambulatory EKG recording.

## Discussion

DS is often found in older adults, with a mean reported age of 57.5 years at the time of initial presentation [13]. The exact mechanism of DS remains unknown. A few pathophysiological explanations have been discussed in the literature. In general, it is postulated that DS is caused by an exaggerated vagal reflex or stimulation initiated by the process of swallowing that results in temporary suppression of cardiac conduction. During swallow, the mechanoreceptors in the esophagus are being stretched and this process sends afferent impulses to the medulla oblongata via the esophageal plexus [14]. The efferent signals are then mediated by the vagal nerves leading to temporary bradyarrhythmia by innervating the sinoatrial and atrioventricular nodes [9]. The parasympathetic response can also result in hypotension secondary to peripheral vasodilation [15]. When it comes to diagnosing DS, it is essential to rule out any esophageal disorders and cardiac diseases beforehand [9]. The diagnosis of DS involves careful history taking and recognition of presyncopal events or syncope triggered by the process of swallowing (provocative test) [13]. It can be further supported by sinus pause on EKG or reduced pulse rate from the baseline during an episode [13], as shown in our case.

Before initial treatment, it is essential to identify and avoid the triggers for the vagal response. Discontinuation of medications that suppress cardiac conduction, such as beta-blockers, is considered the mainstay of therapy [4]. Furthermore, anticholinergic medications and sympathomimetic agents such as atropine have been used in the past to prevent bradyarrhythmia in patients with DS. However, the efficacy of these medications has not been well studied and side effects also limit their use [9]. According to current literature, the definitive management of DS is the insertion of a permanent pacemaker in patients with refractory symptoms despite avoiding triggers, withdrawal from atrioventricular nodal blocking medications, and the addition of sympathomimetic agents [9]. Our patient presented with relatively mild symptoms; therefore, the management started with diet modification and avoidance of dry or coarse food such as tough meats. She remained compliant with these changes as they have helped to prevent symptoms.

## Conclusions

DS is a rare cause of syncope with limited cases in the literature and should be considered part of the diagnostic workup in unexplained syncope. It is imperative to rule out esophageal disorders and cardiac disease before making the diagnosis of DS. Given that patients with DS can develop life-threatening bradyarrhythmia and that it is a difficult condition to diagnose, a better understanding of disease manifestation can help prevent delayed treatment.
